# Canine Bacterial Endocarditis: A Text Mining and Topics Modeling Analysis as an Approach for a Systematic Review

**DOI:** 10.3390/microorganisms12061237

**Published:** 2024-06-19

**Authors:** Annalisa Previti, Vito Biondi, Annamaria Passantino, Mehmet Erman Or, Michela Pugliese

**Affiliations:** 1 Department of Veterinary Sciences, University of Messina, 98168 Messina, Italy; annalisa.previti1@unime.it (A.P.); vito.biondi@unime.it (V.B.); michela.pugliese@unime.it (M.P.); 2 Faculty of Veterinary Medicine, İstanbul University-Cerrahpasa, 34098 Istanbul, Turkey; ermanor@istanbul.edu.tr

**Keywords:** bacterial endocarditis, bartonella, text mining, topic analysis, machine learning

## Abstract

Bacterial endocarditis (BE) is a severe infection of the endocardium and cardiac valves caused by bacterial agents in dogs. Diagnosis of endocarditis is challenging due to the variety of clinical presentations and lack of definitive diagnostic tests in its early stages. This study aims to provide a research literature analysis on BE in dogs based on text mining (TM) and topic analysis (TA) identifying dominant topics, summarizing their temporal trend, and highlighting any possible research gaps. A literature search was performed utilizing the Scopus^®^ database, employing keywords pertaining to BE to analyze papers published in English from 1990 to 2023. The investigation followed a systematic approach based on the PRISMA guidelines. A total of 86 records were selected for analysis following screening procedures and underwent descriptive statistics, TM, and TA. The findings revealed that the number of records published per year has increased in 2007 and 2021. TM identified the words with the highest term frequency-inverse document frequency (TF-IDF), and TA highlighted the main research areas, in the following order: causative agents, clinical findings and predisposing factors, case reports on endocarditis, outcomes and biomarkers, and infective endocarditis and bacterial isolation. The study confirms the increasing interest in BE but shows where further studies are needed.

## 1. Introduction

Infective endocarditis is defined as severe infection involving endocardium and cardiac valves for invasion by infectious agents [1,2,3,4,5]. Although it is rare in dogs, it often results in serious morbidity associated at a mortality rate of up to 30% [6]. Dogs that develop endocarditis show a combination of clinical signs often nonspecific, including depression, weakness, lethargy, weight loss, anorexia, intermittent or shifting lameness, and/or encephalopathy [4,7]. When the congestive heart failure is present, tachypnea, dyspnea, and coughing reflecting left-sided involvement may also occur [1,2,3,4,5,8]. Diagnosis of endocarditis in dogs is challenging due to the variety of clinical presentations, rapid progression, and the limited availability of definitive diagnostic tests for bacterial endocarditis (BE) in the early stages of the disease.

Data mining techniques represent innovative tools, thanks to which data may be simply stored and processed with the support of digital media. TM, also known as text data mining or text analytics, is an advanced technology tuning to turn unstructured data as texts into structured numerical data. TM explores and analyzes large amounts of unstructured data, reducing the potential mistakes, saving time, and providing detailed information from examined texts [9].

The objective of this paper is to provide a complete systematic review using text mining (TM) and topic analysis (TA) techniques applied to the current literature to identify prominent topics and trends correlated to canine BE, and the disease’s pathophysiology, clinical signs, diagnosis, and treatment are also considered.

## 2. Materials and Methods

### 2.1. Literature Search and Descriptive Statistics

A systematic scientific literature search was performed to identify relevant articles with at least an English abstract focused on bacterial endocarditis in dogs. The bibliometric database of Elsevier^©^, i.e., Scopus^®^, was selected due to its accessibility and comprehensive coverage of academic literature [10]. The search was conducted on the 20th of March 2024 and was refined based on the year of publication (from 1900 to 2024), article type (review and scientific article), language (English), and availability of abstract. The keywords used were “endocarditis AND bacterial AND dog”, “endocarditis AND bacterial AND dog AND echocardiography”, “endocarditis AND bacterial AND dog AND congestive AND heart AND failure”, “endocarditis AND bacterial AND dog AND mitral AND regurgitation”, “endocarditis AND bacterial AND dog AND aortic AND insufficiency”, “endocarditis AND bacterial AND dog AND valvular”, “infective AND vegetative AND endocarditis AND dog”, and “cardiac AND infection AND bacterial AND dog”.

A Microsoft Office Excel (version n.16, Microsoft Office 365, Microsoft Corporation, Washington, DC, USA)^®^ spreadsheet containing all records published was retrieved from Scopus^®^. In the database, each line reported a document, and each column provided different information, such as authors, affiliations, abstracts, the year of publication, the type of document (e.g., research article or review), the source of publication (e.g., journal title), and the topic. The records were then screened, and those that did not have information about the abstract, author name, or English text, or were present as duplicates, were automatically excluded. Finally, a manual screening based on the topic and the species discussed in each record to decide the eligibility of the record for inclusion in the final analysis was performed by the authors (APr, MP, VB, and AP). Records related to other species and/or in vitro studies, animal model studies, studies on dog bites, reviews on infectious diseases, reviews on inflammatory diseases, other cardiac diseases, studies on emergency and critical care, and studies on medical treatments were excluded.

Preliminarily, descriptive statistics were conducted to create a detailed profile of the scientific dataset, focusing on the year and journal of publication. Pivot tables were employed to determine the yearly document count and to highlight the leading journals playing a significant role in the issue.

### 2.2. Text Mining Analysis

The articles selected were analyzed using Rstudio for text mining in R (Version 1.3.1093, Free Software Foundation, Boston, MA, USA) following the download process. A dedicated Excel spreadsheet was prepared with two individual columns. The first column called “doc_id” contained the sequential numbering of n.86 documents, while the second column called “text” included abstracts of papers retained for text mining (TM) analysis. The corpus of documents was submitted to pre-processing steps, as reported in the literature [11]. Exactly, the text was converted to lowercase; unusual symbols (such as “@”, “/”, or “*”), punctuation, numbers, and stop words (e.g., “the”, “a”, “and”, “on”, etc.) were removed. In addition, the researchers removed words strictly related to the researched topic or commonly used, such as “dog”, “endocarditis”, “cardiac”, “bacteri”, “dogs”, and “infect”. Extra white spaces that occurred from previous steps were also excluded. In order to reduce words to their root forms, text tokenization was performed.

Thereafter, a document-term matrix (DTM) was built, aligning documents along the rows and terms along the columns. A term frequency-inverse document frequency (TF-IDF) technique was applied to assign relative weights to words, considering both their frequency within a document and prevalence across the document collection. This adjustment enhanced the evaluation of a word’s significance within the document set. Relevant words (TF-IDF ≥ 0.8) were visualized in histograms.

Moreover, a word cloud representing the most relevant words was generated using the website “https://www.wordclouds.com/ (accessed on 5 March 2024)”, where larger character sizes indicated higher TF-IDF values. Associations between the most relevant words (TF-IDF > 1) and all document terms in the corpus were identified based on a correlation threshold of ≥0.2. The statistical analysis was conducted using R packages (2017) and functions from “tm”, “SnowballC”, “ggplot2”, “dplyr”, and “tidyverse.”

### 2.3. Topic Analysis

For the topic modeling analysis, the Latent Dirichlet Allocation (LDA) method was applied. LDA is a hierarchical Bayesian probabilistic approach [12] that identifies thematic topics from words tending to occur together in texts. Each single topic is represented as a multinomial distribution of words, and each single text as a multinomial distribution of latent topics. By analyzing the observed texts and words, the model uncovers the underlying topic structure, generating topic distributions for each text and word distributions for each topic [13,14].

The LDA function with the Gibbs sampling option of the “topic models” package in R was used [15]. The most common words for each topic and their relative probabilities using the “tidytext” R library were visualized. Before starting the analysis, it had to determine the number of topics to split the corpus into. Since the optimal number of topics is generally unknown, it was experimented with 4, 5, 6, and 7 topics, selecting the most informative set based on consensus.

After settling on five topics, they were named with indicative labels. To classify the topics, the cumulative probabilities of the top 10 words in each topic were calculated, and the topics were presented based on this ranking. Each topic was depicted in a bar histogram, with each bar representing the probability of a word within that topic (measured by the beta-value coefficient). This visualization method, in accordance with a previous study [16], assigned a name to each topic for easier identification.

## 3. Results

### 3.1. Descriptive Statistics

**Figure 1:**
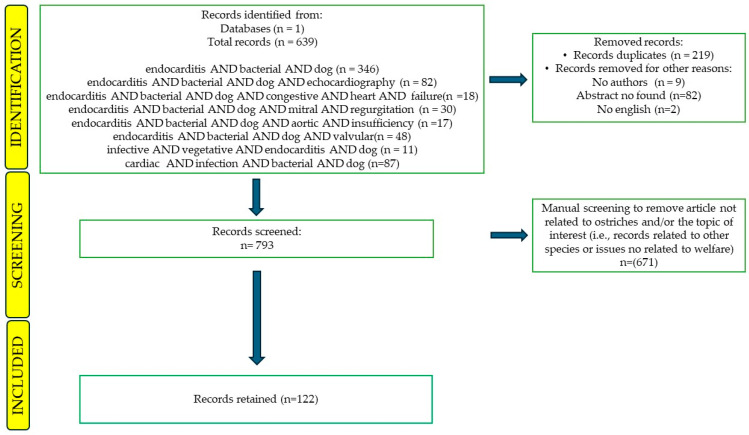
Flow diagram of the review process according to the PRISMA statement.

The literature search retrieved a total of n. 639 records that were filtered in a screening process. The flowchart (Figure 1) illustrates differents steps of the process, showing the number of records that were either kept for further analysis or removed from consideration. Records that posed challenges in categorization underwent review by an expert (MP) who had the authority to determine definitively their inclusion or exclusion from the study (Table 1).

**Figure 2:**
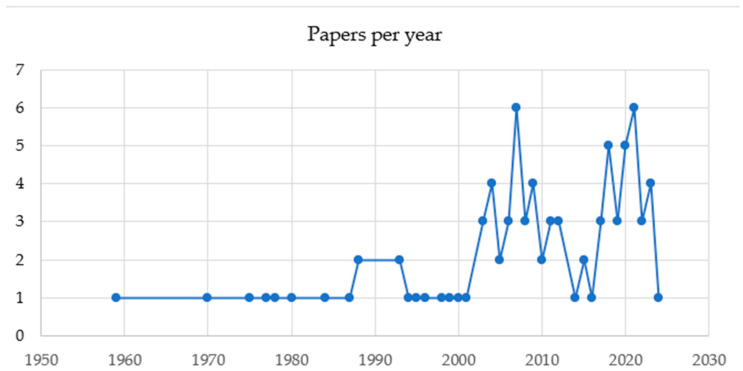
The total number of records published per year between 1950 and 2023.

**Figure 3:**
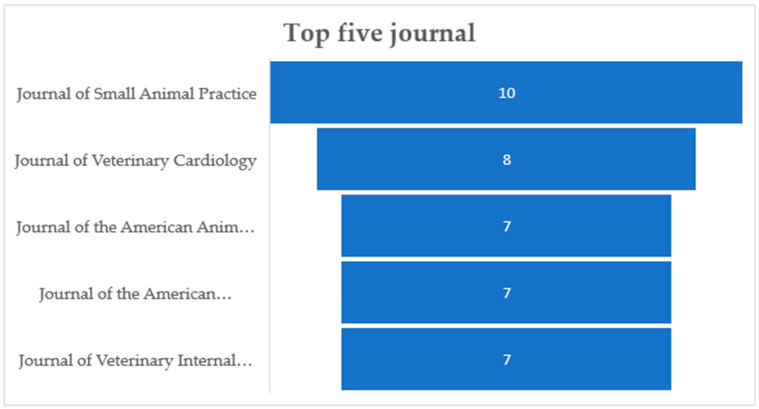
Five journals more representative for the publication of articles related to the topic.

Out of n. 639 abstracts downloaded by Scopus, a total of 86 (13.46%) fulfilled the screening and eligibility criteria and were retained. Articles about other species and/or other topics such as in vitro studies, animal model studies, studies on dog bites, reviews on infectious diseases, reviews on inflammatory diseases, other cardiac diseases, studies on emergency and critical care, and studies on medical treatments (37.71%; n = 241) were excluded. Other reasons for exclusions included the presence of duplicates (34.27%; n = 219), no abstract (12.83%; n = 82), no author found (1.4%; n = 9), and a full text not in English (0.31%; n = 2). The type of records retained were research articles (80/86; 93.02%) and reviews (6/86; 6.98%). The total number of records published per year has increased in 2007 and 2021 (Figure 2). The records were published in 36 different scientific journals of which those with more than n.5 articles on the subject were “Journal of Small Animal Practice” (with 10/86 records; 11.63%), “Journal of Veterinary Cardiology” (n = 8/86 records; 9.3%) and “Journal of the American Animal Hospital Association” (n = 7/86 records; 8.14%), “Journal of the American Veterinary Medical Association”, “Journal of the American Animal Hospital Association”, and “Journal of Veterinary Internal Medicine” (n = 7/86 records; 8.14%) (Figure 3).

Breitschwerdt et al. (1995), focusing on aortic and mitral valvular endocarditis due to *Bartonella vinsonii* subsp. *berkoffii* [17], was the first most cited publication with n.196 citations. MacDonald et al. (2004) [18], who discussed the prevalence of endocarditis induced by *Bartonella* in dogs in northern California, was the second most cited article with n.134 citations. The third most cited articles were Chomel et al. (2009) [19] and Kordick et al. (1996) [20] presented in ex equo n.117 citations. Specifically, Chomel et al. reported a case of endocarditis caused by *B. clarridgeiae* and *B. vinsonii* subsp. *berkoffii* endocarditis in a dog with perforation of the mitral valve, while Kordick et al. focused on two bacterial strains of Bartonella. The fourth most cited article, the most cited in the last 10 years, was Álvarez-Fernández et al. (2018) [21] regarding a European viewpoint on Bartonella endocarditis in a dog. Previous studies about Bartonella infection were mainly performed in North America, so limited data in Europe were available. Data are summarized in Table 2.

### 3.2. Text Mining

**Figure 4:**
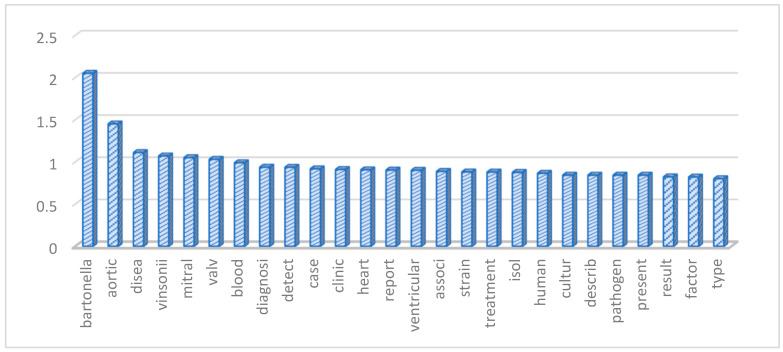
The histogram reports in the root the most frequently words used based on the weighting system (TF-IDF ≥ 0.8).

**Figure 5:**
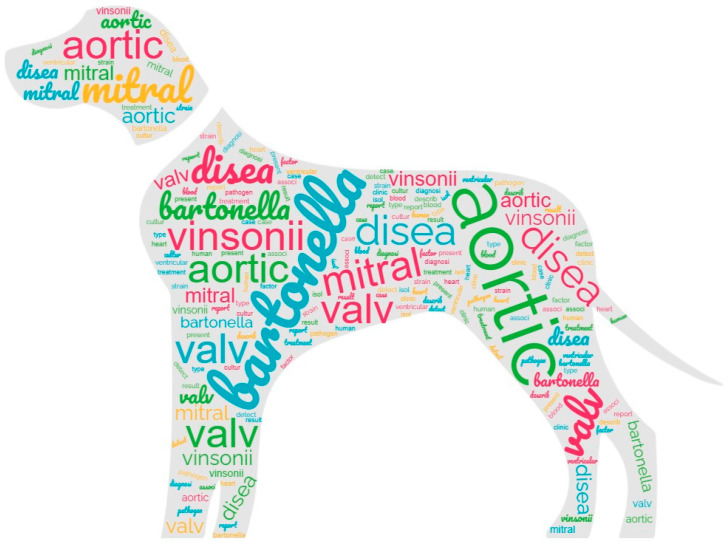
The word cloud reports the words more frequently used. The font size corresponds to the TF-IDF value of each word.

Following data pre-processing and sparseness reduction, a total of 1792 terms were preserved from the initial 86 records. The histogram in Figure 4 shows the most significant words (term frequency-inverse document frequency, TF-IDF ≥ 0.8) based on the TF-IDF weighting system. Additionally, Figure 5 illustrates a word cloud where the font size corresponds to the TF-IDF value of each word. The words with the highest TF-IDF were “bartonella” (2.05), followed by “aortic” (1.45), “disea” (1.11), “vinsonii” (1.07), “mitral” (1.05), “valv” (1.03), “blood” (0.99), “diagnosi” (0.937), “detect” (0.936), “case” (0.917), “clinic” (0.91), “heart” (0.906), “report” (0.903), “ventricular” (0.9), “associ” (0.887), “strain” (0.881), “treatment” (0.879), “isol” (0.875), “huma” (0.865), “cultur” (0.843), “describ” (0.842), “pathogen” (0.842), “present” (0.842), “result” (0.822), “factor” (0.82), and “type” (0.8).

The associations between the most relevant words (with TF-IDF ≥0.8) and the remaining words of the matrix including significant correlations (with correlation grade ≥ 0.2) are shown in Table 3.

### 3.3. Topics Analysis

Five topics were chosen as the ideal topics, and labels were assigned to each of them. The name of each topic as well as the number of records contained in each topic and their first year publication are shown in Table 4.

**Figure 6:**
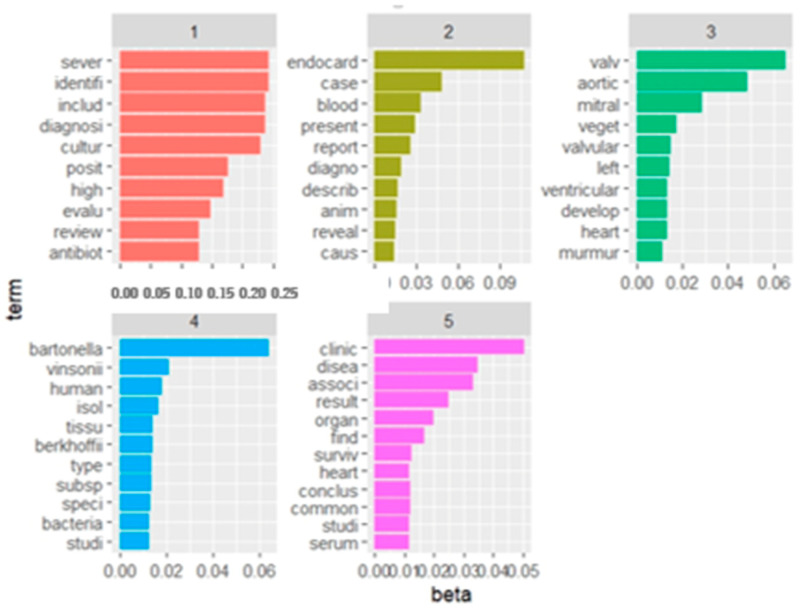
Topics numbered from 1 to 5 according to the cumulative probabilities (CPs), as well as the first 10 words for each topic numbered.

**Figure 7:**
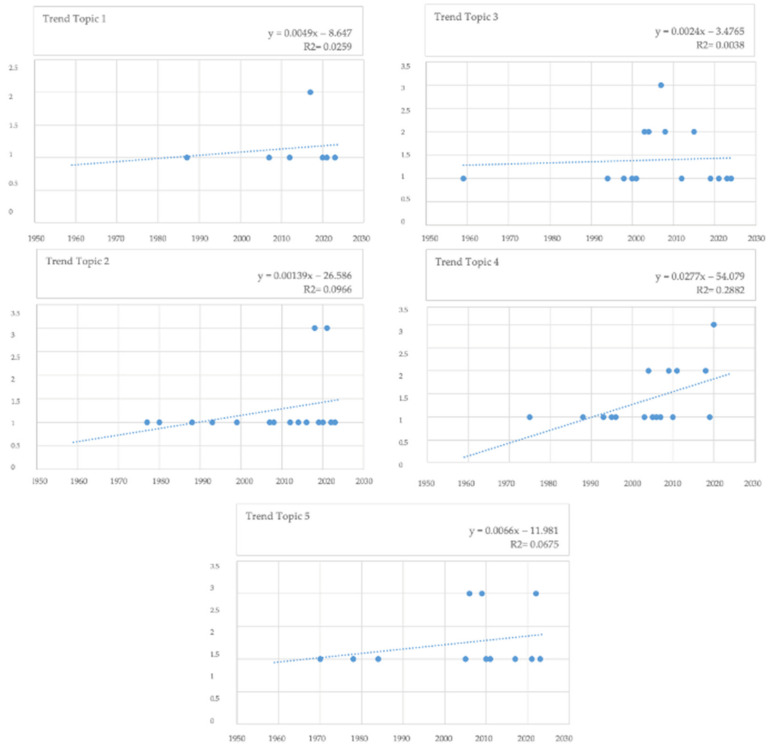
Distribution of the articles within the five topics from 1950 to 2024.

Figure 6 shows the topics numbered from 1 to 5 according to the cumulative probabilities (CPs), as well as the first 10 words for each topic numbered from 1 to 5 according to CPs. Topic 4 (causative agents), Topic 3 (clinical findings and predisposing factors), and Topic 2 (case reports on endocarditis) presented the highest number of records (n.22, 21, and 20 documents, respectively), followed by Topic 5 (outcomes and biomarkers) with n.15 documents and Topic 1 (infective endocarditis and bacterial isolation) with n.8 documents. Figure 7 shows the distribution of the articles within the five topics from 1950 to 2024. A trendline shows an increase of the number of papers published for each topic.

## 4. Discussion

Through the use of advanced machine learning methods such as TM and TA, this study delved into the complexities of BE in dogs by analyzing a diverse selection of scientific literature published since 1900. By employing these methodologies, the authors were able to examine different aspects of BE and identify specific areas where lacks in knowledge are present. The study findings reveal that articles focused on therapeutic approaches are less common compared to those addressing causative agents, markers, or clinical findings.

The number of published articles on BE in dogs has shown an increase starting from 2001, reaching peaks in both 2007 and 2021. This trend is not surprising, given, on one side, the growing carefulness on animal health among pet owners and, on the other side, the improvement of diagnostic capabilities in recent years that has heightened awareness about these infections [4,6].

A comparable rise in BE diagnoses has also been observed in human patients, with an annual increase of 2.4% from 1998 to 2009 [22].

The first 10 words appearing, ranked by their weight and close in their meaning, probably highlight that one of the extensively researched aspects related to BE in dogs is the association between *Bartonella* infection and valvular disease, in particular involving aortic and mitral valves. Among the various terms, it is noteworthy that the word “Bartonella” appears with higher frequency in TM analysis.

Bartonellae, a group of emerging pathogens transmitted by vectors, invade the red blood cells and endothelial cells of diverse domestic and wild mammals [23]. The discovery of *Bartonella vinsonii* subspecies *berkhoffii* in a canine with endocarditis in 1993 marked a significant turning point [17] establishing this microorganism as a crucial pathogen in dogs [19]. The clinical and pathological features of canine endocarditis closely resemble those observed in human patients, with a higher incidence of aortic valve involvement and extensive vegetative lesions accompanied by calcification, and commonly elevated levels of *Bartonella* antibodies [24]. Likely attributed to the correlation with human endocarditis, the term “human” holds a substantial weight (0.865) among the commonly used words in the context of TM.

The prevalence of *Bartonella* associated to endocarditis in dogs is among the highest reported to date [23], with an incidence of 20% to 30% reported in California state [4,19]. Fenimore et al. (2011) [25] revealed that nearly 80% of dogs with a diagnosis of endocarditis were infected with Bartonella in Colorado. Recent findings [23] documented a case of canine cardiac infections caused by *B. washoensis*, previously identified in a California dog. Cases of canine infection with *B. elizabethae* have been documented in the USA [26], Algeria [27], and Thailand [28] with a notable instance in a military working dog imported from Germany. Historical records show occurrences of *B. henselae* endocarditis, endomyocarditis, or endocardiosis in dogs serving in Southeast Asia during the 1970s, with cases linked to *B. vinsonii* subsp. *berkhoffii, B. washoensis*, and *B. elizabethae* documented in dogs that perished in the 1980s across various regions [23]. Bartonella-induced endocarditis in canines typically presents with severe cardiac lesions, particularly valvular vegetative lesions, and a low survival rate [4,19,23].

Other considerations can be made based on the associations between the words. The term “*Bartonella*” is frequently associated with “ectoparasite” and “vector borne”, obviously because *Bartonella* spp. are the etiological agents of several emergent vector-borne diseases, induced by ectoparasites [29], that have a broad spectrum of clinical presentations including endocarditis, granulomatous diseases, meningoencephalitis, polyarthritis, uveitis, or hemolytic anemia [30]. The infective endocarditis is also considered an uncommon “life-threatening” disorder in dogs [31,32].

The association of the term “disease” with “biomarker,” “periodont,” and “immunomediated” deserves some consideration. Despite the severe nature of the disease and the high fatality rate linked to infective endocarditis in canines, establishing a definitive diagnosis can pose challenges before death due to the presence of non-specific clinical symptoms [4]. Given that the diagnosis of endocarditis primarily relies on a scoring system known as the modified Duke criteria [33], any supplementary tests that can contribute to confirming an infective endocarditis diagnosis hold significant value, particularly for veterinarians with limited exposure to this condition [34]. This heightened interest in identifying markers for BE in dogs can be attributed to the complexity of diagnosing the disease accurately. Kilkenny et al. (2021) [34] have demonstrated that cardiac troponin levels serve as a valuable tool in distinguishing dogs with BE. Cardiac troponin I (cTnI) is an intracellular protein found in the myocardium, and elevated levels of this biomarker may indicate localized myocarditis triggered by inflammatory mediators, septic or thrombotic coronary emboli, or direct myocardial involvement by the infection itself [35]. These mechanisms, akin to those observed in humans, have been proposed as potential explanations for the increased cTnI levels in canines with BE [34].

The correlation with the word “periodontal” is not unexpected, given the elevated prevalence of periodontal disease in dogs and their correlation to infective endocarditis. Any connection between periodontal disease and systemic organ damage holds significant importance for canine health. The presence of systemic diseases in dogs with chronic periodontal disease is often linked to bacteremia and/or bacterial toxins in oral cavity [36]. Moreover, there is a notable relationship between the gravity of periodontal disease and the risk that endocarditis develops in a dog [37]. *Actinobacillus actinomycetemcomitans*, a suspected periodontal pathogen, is among the causative microorganisms for infective endocarditis [37]. The way of colonization by infective agents in microscopic sterile lesions remains unclear; nevertheless, in human studies, it has been documented that transient bacteremia frequently arises after periodontal procedures, with periodontal bacteria being identified in atheromatous plaques in patients with chronic periodontitis [38,39,40]. Finally, regarding the association between “disease” and “immunemediat”, BE are influenced by various factors, with the host’s immune response playing a crucial role. Conditions that compromise the immune system, such as immune-mediated diseases, can increase the susceptibility to BE [41,42,43]. Furthermore, there is a possibility that cases of endocarditis caused by erysipelothrix bacteria may lead to subsequent complications, such as secondary immune-mediated hemolytic anemia and thrombocytopenia, a condition known as Evans syndrome [33].

This analysis underscored the main BE-focused research in dogs. The trending topics with the highest numbers of records are strictly correlated, and it is easy to understand their interconnectedness.

The topic with the highest number of published papers on BE was related to causative agents (Topic 4). The papers included within this topic revealed *Bartonella* spp., *Pasteurella,* and *Erysipelothrix tonsillarum*, examples of the most indagate causative agents [43,44,45,46,47,48]. The others most common causative agents are, firstly, Staphylococcal species (including aureus, intermedius, coagulase-positive, and coagulase-negative), followed by Streptococcus species (such as canis, bovis, and beta-hemolytic), and *Escherichia coli*. Less common bacterial isolates associated with IE include Pseudomonas, Erysipelothrix rhusiopathiae, Enterobacter, Pasteurella, Corynebacterium, and Proteus. Rare, isolated microorganisms comprise Bordetella avium-like organism, and *Actinomyces turicensis* [49].

The second most important topic was “Clinical findings and predisposing factors” (Topic 3). The first predisposing factor of BE is the presence of bacteremia and endothelium disruption. Subaortic stenosis stands out as the most prevalent cardiac anomaly in dogs afflicted with BE, leading to turbulent blood flow and injury to aortic cusps [49,50]. While other cardiac conditions have not been statistically linked to an increased risk of BE in dogs, myxomatous valve degeneration emerges as the primary heart disease, particularly affecting small-breed [49]. Various conditions, such as diskospondylitis, prostatitis, pneumonia, urinary tract infections, pyoderma, periodontal disease, and the prolonged presence of central venous catheters, serve as common sources of bacteremia in dogs. The role of immunosuppression as a predisposing factor for infective endocarditis remains unclear within the scientific community. For many years, dental prophylaxis has been suggested as a potential predisposing factor for BE development in dogs based on anecdotal evidence [50]. An interesting association was studied between bacterial cholecystitis and concurrent BE [51]. Finally, the available literature offers scarce documentation on the association between BE and hypertrophic osteophathy. Probably, pulmonary shunting, vagal nerve stimulation, the production of humoral substance by neoplastic cells, and the megakaryocite/platelet clump are involved in its pathogenesis [47]. In a study by Dunn et al. (2007) [47], a case of hypertrophic osteopathy linked with IE in an adult boxer dog was reported, shedding light on this rare occurrence in veterinary medicine. This case study underscores the complexity and diversity of manifestations associated with IE in canines, highlighting the need for further research and understanding in this field.

Regarding clinical findings, a heart murmur is detected in 89–96% of dogs with IE [49]. In patients with bacteremia or sepsis, mucous membranes may exhibit signs of injection, while those with low-output heart failure may present with pale mucous membranes. Tachypnea, dyspnea, cough, or abnormal lung sounds are prevalent, reflecting the high incidence of heart failure (50%) among dogs affected by IE. Fever is a common symptom, observed in 50% to 74% of cases, although it may occur intermittently. Additionally, dogs with IE often display physical abnormalities like lameness, joint pain, and swelling. Neurological manifestations are not rare, affecting 23% of dogs in one study. These abnormalities may include ataxia, impaired conscious proprioception, reduced alertness, cranial nerve deficits, and signs of vestibular dysfunction. Arterial thromboembolism typically occurs most frequently in the right thoracic limb or pelvic limbs, adding to the spectrum of clinical findings associated with IE in dogs [49].

The following most important topics are “Case reports on endocarditis “(Topic 2) and “Outcomes and biomarkers” (Topic 5), and are correlated. It is noteworthy that the majority of the papers relating to IE in dogs are case reports [43,44,45,46,47,48]. The last topic in order of importance was “Infective endocarditis and bacterial isolation” (Topic 1), probably due to the lower interest in bacterial isolation compared with clinical findings in dogs.

The limitations of the methodology employed in this review needs to be underlined. Search strings may not have included all possible synonyms, potentially limiting the scope of records included. Additionally, records outside of the Scopus^®^ database were not considered, which could have influenced the comprehensiveness of the review. Search parameters, such as the requirement for English language abstracts and specific screening criteria, may have further restricted the number of records analyzed. Moreover, the review methodology involved only assessing titles and abstracts of the 86 records, rather than a full reading of each document. Despite these limitations, the study provided valuable insights into canine BE research, highlighting key topics and knowledge gaps.

## 5. Conclusions

This review applied machine learning equipment to investigate and explore the literature concerning BE in dogs. The results revealed a suggestive increase in interest regarding canine infectious endocarditis, reflected by scientific literature focused on general clinical, diagnostic, and laboratory findings in the last decade.

## Figures and Tables

**Table 1:** Inclusion and exclusion criteria applied.

Inclusion criteria (labels)		**Reasons**
	Language	English
	Years	1900–2024
	Topic	Bacterial endocarditis in dog
	Source	Articles and/or Reviews
	Available	Abstract, full text, authors, journal
Exclusion criteria (labels)	Other/many species	Reports relating to other species (feline, humans, rabbits, etc.)
	Experimental studies	Reports relating to in vitro studies and/or animal models studies
	Reviews on other related topics	Reports relating to infectious diseases, reviews on inflammatory diseases, and/or other cardiac diseases; studies on dog bites
	Studies on emergency care	Reports relating to emergency and/or critical care
	Studies on medical treatments	Reports relating to drugs, and/or medical treatment

**Table 2:** The most cited documents.

No.	Authors/Year/Journal	Title of the Publication	GC
1	Breitschwerdt, E.B., et al., 1995, Journal of Clinical Microbiology [17]	Endocarditis in a Dog Due to Infection with a Novel Bartonella Subspecies	196
2	MacDonald, K.A., et al., 2004, Journal of Veterinary Internal Medicine [18]	A Prospective Study of Canine Infective Endocarditis in Northern California (1999–2001): Emergence of Bartonella as a Prevalent Etiologic Agent	134
3	Chomel, B.B., et al., 2009, Annals of the New York Academy of Sciences [19]	*Bartonella endocarditis*: A Pathology Shared by Animal Reservoirs and Patients	117
4	Kordick, D.L., et al., 1996, International Journal of Systematic Bacteriology [20]	*Bartonella vinsonii* subsp. *berkhoffii* subsp. nov., Isolated from Dogs, *Bartonella vinsonii* subsp. *vinsonii*, and an Emended Description of *Bartonella vinsonii*	117
5	Álvarez-Fernández, A., et al., 2018, Parasites and Vectors [21]	Bartonella Infections in Cats and Dogs Including Zoonotic Aspects	104

**Table 3:** Associations between the most relevant words (with TF-IDF ≥0.8) and the remaining words of the matrix.

Words (TF-IDF ≥ 0.1)	Associated Words (Correlation Grade ≥ 0.2)
Bartonella	Ectoparasite (0.53); lifethreaten (0.53); sequenc (0.53); hensela (0.51); gene (0.50); titer (0.49); restrict (0.47); glta (0.46); speci (0.46); Chilean (0.45); country (0.45); freeroam (0.45); half (0.45); heavi (0.45); linar (0.45); montt (0.45); puerto (0.45); seroprev (0.45); south (0.45); stray (0.45); infest (0.44); California (0.43); chain (0.43); june (0.43); polymera (0.43); American (0.42); rang (0.41); vectorborn (0.41)
Aortic	Coronaric (0.48); thougth (0.41)
Disease	Cohort (0.59); agematch (0.57); cardiovascularrel (0.57); comparison (0.57); confound (0.57); connect (0.57); designhistor (0.57); estim (0.57); flora (0.57); marker (0.57); model (0.57); noncardiovascularrel (0.57); nonperiodont (0.57); objectiveto (0.57); periodont (0.57); priority (0.57); probabl (0.57); procedurescox (0.57); relevanceth (0.57); resultssignif (0.57); immunemedi (0.55); stage (0.53); proport (0.51); broadspectrum (0.50); contribut (0.50); difficulti (0.50); longterm (0.50); underreport (0.50); worst (0.50); cardiomyopathi (0.49); major (0.45); preval (0.42); higher (0.41)
*Vinsonii*	berkhoffii (0.87); subspeci (0.86); diver (0.59); effort (0.59); elucid (0.59); meningoenceph (0.59); vascul (0.59); acccommod (0.55); atcc (0.55); believ (0.55); closest (0.55); composit (0.55); emend (0.55); granulomat (0.55); indic (0.55); relat (0.55); rrnas (0.55); separ (0.55); neutrophil (0.54); propo (0.54); repeat (0.54); recogn (0.53); seroreact (0.53); manifest (0.52); subsp (0.52); level (0.51); insert (0.50); uveitis (0.49); potenti (0.47); rrna (0.47); titer (0.47); antibody (0.46); becam (0.41); sequenc (0.41).
Mitral	Fibrosa (0.50); normal (0.46); intact (0.43); holosystol (0.42); concern (0.41); former (0.41); haemodynam (0.41); incompet lipoid (0.41); mucoid (0.41); nodular (0.41); stimuli (0.41); synopsi (0.41)
Valv	California (0.47); antemortem (0.46); clarridgeiaelik (0.46); longer (0.46); northern (0.46); frlp (0.46); postmortem (0.41)

**Table 4:** Different topics examined and the number of records contained with the first year of publication in each topic.

Topic Number	Label of the Topic	Papers (*n*)/from Year
1	Infective endocarditis and bacterial isolation	8/1987
2	Case reports on endocarditis	20/1977
3	Clinical findings and predisposing factors	21/1959
4	Causative agents	22/1975
5	Outcomes and biomarkers	15/1970

## Data Availability

No new data were created or analyzed in this study.
